# *Thymus zygis* subsp. *zygis* an Endemic Portuguese Plant: Phytochemical Profiling, Antioxidant, Anti-Proliferative and Anti-Inflammatory Activities

**DOI:** 10.3390/antiox9060482

**Published:** 2020-06-03

**Authors:** Amélia M. Silva, Carlos Martins-Gomes, Eliana B. Souto, Judith Schäfer, João A. Santos, Mirko Bunzel, Fernando M. Nunes

**Affiliations:** 1 Department of Biology and Environment, School of Life Sciences and Environment, University of Trás-os-Montes and Alto Douro (UTAD), 5001-801 Vila Real, Portugal; 2 Centre for Research and Technology of Agro-Environmental and Biological Sciences (CITAB), UTAD, 5001-801 Vila Real, Portugal; camgomes@utad.pt (C.M.-G.); jsantos@utad.pt (J.A.S.); 3 Food and Wine Chemistry Lab., Chemistry Research Centre-Vila Real (CQ-VR), UTAD, 5001-801 Vila Real, Portugal; 4 Department of Pharmaceutical Technology, Faculty of Pharmacy, University of Coimbra, Pólo das Ciências da Saúde, 3000-548 Coimbra, Portugal; ebsouto@ff.uc.pt; 5 CEB–Centre of Biological Engineering, University of Minho, Campus de Gualtar, 4710-057 Braga, Portugal; 6 Department of Food Chemistry and Phytochemistry, Institute of Applied Biosciences, Karlsruhe Institute of Technology (KIT), Adenauerring 20a, Building 50.41, 76131 Karlsruhe, Germany; judith.schaefer@kit.edu (J.S.); mirko.bunzel@kit.edu (M.B.); 7 Department of Physics, School of Sciences and Technology, UTAD, 5001-801 Vila Real, Portugal; 8 Department of Chemistry, School of Life Sciences and Environment, UTAD, 5001-801 Vila Real, Portugal

**Keywords:** *Thymus zygis* subsp. *zygis*, phenolic profiling, aqueous decoction, hydroethanolic extract, luteolin-*O*-hexoside, anti-proliferative activity, radical scavenging activity, antioxidant, anti-inflammatory activity

## Abstract

*Thymus zygis* subsp. *zygis* is an endemic Portuguese plant belonging to the *Thymus zygis* species. Although *T. zygis* is commonly used as a condiment and as a medicinal herb, a detailed description of the polyphenol composition of hydroethanolic (HE) and aqueous decoction (AD) extracts is not available. In this work, we describe for the first time a detailed phenolic composition of *Thymus* *zygis* subsp. *zygis* HE and AD extracts, together with their antioxidant, anti-proliferative and anti-inflammatory activities. Unlike other *Thymus* species, *T.* *zygis* subsp. *zygis* extracts contain higher amounts of luteolin-(?)-*O*-hexoside. However, the major phenolic compound is rosmarinic acid, and high amounts of salvianolic acids K and I were also detected. *T.* *zygis* subsp. *zygis* extracts exhibited significant scavenging activity of ABTS^+^, hydroxyl (^•^OH), and nitric oxide (NO) radicals. Regarding the anti-proliferative/cytotoxic effect, tested against Caco-2 and HepG2 cells, the AD extract only slightly reduced cell viability at higher concentrations (IC_50_ > 600 µg/mL, 48 h exposure), denoting very low toxicity, while the HE extract showed a high anti-proliferative effect, especially at 48 h exposure (IC_50_ of 85.01 ± 15.10 μg/mL and 82.19 ± 2.46 μg/mL, for Caco-2 and HepG2, respectively). At non-cytotoxic concentrations, both extracts reduced the nitric oxide (NO) release by lipopolysaccharide (LPS)-stimulated RAW 264.7 cells (at 50 μg/mL, HE and AD extracts inhibited NO release in ~89% and 48%, respectively). In conclusion, the results highlight the non-toxic effect of aqueous extracts, both resembling the consumption of antioxidants in foodstuff or in functional food. Furthermore, the HE extract of *T.* *zygis* subsp. *zygis* is a source of promising molecules with antioxidant, anti-inflammatory and anticancer activities, highlighting its potential as a source of bioactive ingredients for nutraceutical and pharmaceutical industries.

## 1. Introduction

The genus *Thymus*, belonging to the Lamiaceae family, includes ca. 350 species of perennial, subshrubs, and aromatic herbs native to Europe and North Africa, with many of them being endemic to the Mediterranean region [1,2,3]. *Thymus* plants are heliophylous, thus they grow well in a climate with moderate to warm temperatures, in well-drained to dry soils (usually they grow on rocks, stones, or sand), and in sunny places [4]. Besides these ecological preferences, some *Thymus* species are easily cultivated, especially in calcareous light, dry, stony soils and heavy wet soils, but lose some aromatic properties [4]. 

*Thymus zygis* Loefl. ex L. (Lamiaceae) grows in the countries around the Mediterranean Sea and is widespread throughout Portugal and Spain [5,6]. *Thymus zygis* is commonly named “erva-de-Santa-Maria”; “sal-da-terra”, “serpão-do-monte” (Portuguese), white-thyme, and others [7,8]. For this species, three subspecies are described, namely *Thymus zygis* subsp. *zygis* Loefl. ex L.; *Thymus zygis* subsp. *gracilis* (Boiss.) R. Morales, and *Thymus zygis* subsp. *sylvestris* (Hoffmanns. & Link) Coutinho [1,9]. In Portugal, only two of these subspecies are found, *T. zygis* subsp. *zygis* and *T. zygis* subsp. *sylvestris*, which present differences in some botanical characteristics, chromosome number, and ecology [6,9]. However, in Spain, it is possible to find *T. zygis* subsp. *gracilis*, instead of *T. zygis* subsp. *zygis* [5,10]. In Portugal, *T. zygis* L. subsp. *sylvestris* is commonly found in the central regions, and it is traditionally used in the preservation of food (e.g., olives), as a condiment (e.g., in cheese, fish, meat, salads, sauces), as a digestive tonic, and in the treatment of colds and sore throat [11]. *T. zygis* subsp. *gracilis* (known as red thyme) essential oil is rich in thymol, and due to its relevance in thyme essential oil quality, *T. zygis* has become the most commercialized thyme in Spain because of its economic importance [12]. Indeed, *T. zygis* is amongst the five thyme species with the highest commercial value, together with *Thymus vulgaris* L. (common thyme), *Thymus capitatus* (L.) Hoffmanns. et Link (recently classified as *Thymbra capitata* (L.) Cav.), *Thymus mastichina* L., *Thymus serpyllum* L., mostly due to the essential oils, but *T. vulgaris* and *T. zygis* have also high economic values for culinary and seasonings, mostly sold as dry herbs [1]. 

Although the essential oil composition of the different *T. zygis* subspecies has been thoroughly described in the literature [6,12,13,14], together with the related bioactive properties [11,13,15], studies concerning its polyphenol composition and bioactivity are scarcer. The methanolic and ethyl ether extracts of *T. zygis* were however shown potent antioxidant activity resulting from a direct correlation with their phenolic content [16,17]. As far as we known, only three studies report the polyphenolic composition of *T. zygis*, with one of these being performed in *T. zygis* (subspecies *gracilis*) hydrodestillation by-products aiming the valorization of this abundant waste [18]. In other study, water extraction of *T. zygis* (subspecies not described) polyphenols, mimicking a decoction preparation, revealed rosmarinic acid as the major phenolic compound, and showed antioxidant and anti-bacterial activities towards Gram-positive *Staphylococcus aureus* and *Staphylococcus epidermidis* and Gram-negative *Escherichia coli*, *Salmonella typhimurium* and *Pseudomonas aeruginosa* bacteria [19]. In the third study, aqueous extracts of *T. zygis* subsp. *gracilis*, rich in caffeic and rosmarinic acids, revealed moderate to high antioxidant activity, potent anti-inflammatory activity (in a mice model of croton oil-induced ear edema), and strong anticoagulant activity [20]. Anti-inflammatory, antioxidant, and anticoagulant effects were also described for non-characterized *T. zygis* aqueous extracts [21]. In a study involving several medicinal plants, methanolic extracts of *T. zygis* (the three subspecies) showed potent anti-bacterial activity against the Gram-positive *Staphylococcus aureus* and *Bacillus cereus*, which was correlated with the high total phenolic content (quantified with Folin–Ciocalteau’s reagent) obtained for these extracts [17].

Although few research articles refer to the *T. zygis* phenolic composition, none offered a complete/exhaustive description. *T. zygis*, together with *T. vulgaris*, is mentioned by the European Medicines Agency (EMA) through the Committee on Herbal Medicinal Products (HMPC), and approved in several pharmaceutical preparation forms, in which water and ethanol are the main recommended solvents [22,23]. Indeed, *T. zygis* products (extracts and/or essential oils) are used as an ingredient in several pharmaceutical and dietary supplement preparations, such as anti-cough syrups (e.g., Sideri-Bronsid, from Sideri Laboratory, Belgium; Pertusinas^®^ Forte, from VALENTIS, Lithuania), anti-cough pastilles (e.g., Buttercup Bronchostop Cough Pastilles; Omega Pharma Ltd., London, UK), expectorant and anti-cough syrups (e.g., hydraSense^®^ Mucus & Phlegm Cough Syrup, from Bayer AG, Leverkusen, Germany), mouth and throat sprays (e.g., LaDrôme Propolis throat and mouth spray, Ladrôme Laboratoire, Saillans, France), and others.

Another important field of *T. zygis* application is in the livestock and food industry. The inclusion of *T. zygis* subsp. *gracilis* leaves in the diet of pregnant sheep was reported to positively affect the sensorial characteristics, as well as the oxidative stability of cooked lamb meat [24]. Goats fed with distilled and non-distilled *T. zygis* subsp. *gracilis* leaves showed an improvement in the sensory (reduced lipid oxidation) and nutritional properties (increased content in protein, fat, dry matter, and polyunsaturated fatty acids) of milk, as well as of cheese [25]. Rabaçal cheese (PDO, protected designation of origin), produced in central Portugal where sheep and goat are fed freely in fields rich in *T. zygis,* has a distinctive characteristic aroma and flavor, attributed to the thyme [26]. To the best of our knowledge, there is no scientific report in which the presence of *T. zygis* bioactive compounds was characterized/quantified in Rabaçal cheese. 

As the phenolic composition of *T. zygis*, and especially that of *T. zygis* subsp. *zygis*, is not fully known, due to its potential bioactivities and economic value, the main objective of this work was to determine the polyphenol composition of *T. zygis* subsp. *zygis* hydroethanolic extracts, by applying an exhaustive extraction procedure to access the whole extractable polyphenol composition, and aqueous decoction extracts, mimicking the human consumption as herbal tea. Together with the phenolic profile of *T. zygis* subsp. *zygis* extracts, this work also aims to characterize the extract’s anti-oxidant activity, anti-proliferative/cytotoxic activity against Caco-2 and HepG2 cell lines, and the anti-inflammatory activity, using the LPS-stimulated RAW 264.7 cell model.

## 2. Materials and Methods

### 2.1. Standards and Reagents

Methanol (HPLC or MS grade), ethanol, formic acid, acetic acid, hydrogen peroxide (30% solution), trichloroacetic acid (TCA), Folin–Ciocalteau’s reagent, 2-deoxy-D-ribose, sodium nitrite, sodium nitroprusside, potassium persulfate, sodium molybdate, aluminum chloride (III), ethylenediaminetetraacetic acid (EDTA), ascorbic acid, sulfanilamide, *N*-(1-naphthyl)ethylenediamine dihydrochloride, 2,2-azino-bis (3-ethylbenzothiazoline-6-sulfonic acid) diammonium salt (ABTS), (±)-6-hydroxy-2,5,7,8-tetramethylchromane-2-carboxylic acid (Trolox), thiobarbituric acid (TBA), and standards of rosmarinic acid, catechin, luteolin, apigenin, and ursolic acid were purchased from Sigma-Aldrich/Merck (Algés, Portugal). Caffeic acid was obtained from Extrasynthese^®^ (Genay, France). Oleanolic acid was obtained from Santa Cruz Biotechnology Inc. (Frilabo; Porto, Portugal). Dulbecco’s Modified Eagle Medium (DMEM), sodium pyruvate, penicillin, streptomycin, versene, L-glutamine, trypsin-EDTA, and foetal bovine serum (FBS) were obtained from Gibco (Alfagene, Invitrogen, Portugal). Alamar Blue^®^ was obtained from Invitrogen, Life-Technologies (Porto, Portugal).

### 2.2. Plant Material

*T. zygis* subs *zygis* (*T. zygis*) aerial parts (upper part of stems, their leaves and flowers) were grown in organic farming conditions and harvested in April 2016 (beginning of flowering stage) in Mezio, Viseu, Portugal; at 40°58’47.4” N 7°53’43.3” W and supplied by ERVITAL^®^ (Plantas Aromáticas e Medicinais, Lda). A voucher specimen (*T. zygis* subsp. *zygis*: Voucher N. HVR21092) was deposited in the botanical garden office at the University of Trás-os-Montes and Alto Douro (UTAD, Vila Real, Portugal) after authentication. Immediately after the harvest, the plant material was rinsed with distilled water, weighted, and frozen (−20 °C). Plants were lyophilized (Dura Dry TM μP freeze-drier; −45 °C, 250 mTorr) and then were conveniently stored. 

### 2.3. Preparation of Extracts

Freeze-dried *T. zygis* aerial parts were ground to a fine powder (using a coffee mill) and then extracted according to two extraction methods: aqueous decoction (AD), aiming to mimic human consumption as a herbal tea or condiment, and exhaustive hydroethanolic extraction (HE), a method optimized to obtain all the extractable compounds within the plant material, as described in Martins-Gomes et al. [27]. For both extraction methods, 0.5 g of lyophilized and ground plants were used. For the AD extraction, 150 mL of distilled water were added to the plant material and boiled for 20 min, under constant stirring. The extract was filtered twice (Whatman n° 4 filter, Whatman, USA, and fiberglass filter 1.2 μm; VWR International Ltd., Radnor, PA, USA). For the HE extraction, 50 mL of ethanol solution (80% *v*/*v*, in water) were added to the plant fine powder. The mixture was agitated at room temperature for one hour (orbital shaker, 150 rpm) and centrifuged (7000 rpm, 4 °C; for 5 min, Sigma Centrifuges 3–30 K, St. Louis, MO, USA). After centrifugation, the supernatant was filtered twice (Whatman n° 4 filter, Whatman, USA; and fiberglass filter, 1.2 μm, VWR International Ltd., Radnor, PA, USA) and collected. Then extraction of the pellet was repeated two times more as described above. All the supernatants were combined. Both extracts were concentrated in a rotary evaporator (35 °C), freeze-dried, weighted to calculate the yields, and properly stored until further analysis.

### 2.4. Total Phenolic Compound Content

For the determination of the total phenolic compound (TPC) contents of the extracts the Folin–Ciocalteau method was used. To 1 mL of *T. zygis* extracts (0.1 mg/mL), 0.5 mL of Folin–Ciocalteau reagent, and 1 mL of sodium carbonate (Na_2_CO_3_; 7.5 %, *w*/*v*) were added, and the volume was adjusted to 10 mL with distilled water. The mixture was incubated (1 h, room temperature), and the absorbance at 725 nm was read (PerkinElmer, Lambda 25 UV/VIS Spectrometer) [28]. Caffeic acid was used as standard and TPC was expressed as caffeic acid equivalents (mg CA eq./g lyophilized plant or mg CA eq./g extract) [29,30].

### 2.5. Total Flavonoid Content

For the determination of the total flavonoid content (TFC) of the extracts, the method described by Jia et al. [31] was used. To 1 mL of *T. zygis* extracts solution (0.5 mg/mL), 150 μL of an aqueous sodium nitrite solution (NaNO_2_; 5%, *w*/*v*) was added, and the mixture was incubated at room temperature for 5 min. After this time, 150 μL of AlCl_3_ solution (10 %, *w*/*v*) were added and incubated for 6 min. Finally, 1 mL of sodium hydroxide solution (NaOH; 1 M) was added and the absorbance at 510 nm was read. The used standard was catechin, and TFC was expressed as mg catechin equivalents (mg C eq./g lyophilized plant or mg C eq./g extract).

### 2.6. Total Ortho-Diphenol Content

For the determination of the *ortho*-diphenol (ODP) content, the method described by Machado, Felizardo, Fernandes-Silva, Nunes and Barros [28] was used. To 4 mL of the *T. zygis* extracts (0.1 mg/mL), 1 mL of sodium molybdate solution (Na_2_MoO_4_; 5%, *w*/*v*) was added, and the mixture was incubated at room temperature for 15 min. The absorbance was read at 370 nm. Caffeic acid was the standard used, and the ODP content was expressed as mg caffeic acid equivalents (mg CA eq./g lyophilized plant or mg CA eq./g of extract).

### 2.7. In Vitro Antioxidant Activity Assessment

#### 2.7.1. ABTS^•+^ Scavenging Assay

The ABTS^•+^ scavenging activity of *T. zygis* extracts was measured using the method described by Machado, Felizardo, Fernandes-Silva, Nunes and Barros [28]. ABTS^•+^ was produced by mixing equal volumes of a 7 mM aqueous ABTS solution and a 2.45 mM solution of potassium persulfate. The mixture was allowed to react at room temperature in the dark for 15–16 h. After this time, the mixture was diluted in 20 mM acetate buffer at pH 4.5, in order to obtain an absorbance at 734 nm of 0.700 ± 0.02. The scavenging activity of *T. zygis* extracts were measured by adding 2 mL of the diluted ABTS^•+^ solution to 200 μL of extracts (0.1 mg/mL). The mixture was incubated for 15 min at room temperature, and the absorbance at 734 nm was read. The antioxidant standard used was Trolox ((±)-6-hydroxy-2,5,7,8-tetramethylchromane-2-carboxylic acid). The ABTS^•+^ scavenging activity was expressed as Trolox equivalents (mmol Trolox/g lyophilized plant or mmol Trolox/g extract).

#### 2.7.2. Hydroxyl Radicals Scavenging Assay

For the determination of the site-specific and non-site-specific hydroxyl radical (^•^OH) scavenging activity, the methods described by Taghouti et al. [32] were used. For the site-specific assay, to 0.5 mL of *T. zygis* extracts extract solution (0.1 mg/mL), 100 μL of deoxyribose (20 mM), 100 μL of iron (II) chloride solution (FeCl_2_; 1 mM), 100 μL of ascorbic acid solution (1 mM), and 100 μL of hydrogen peroxide (H_2_O_2_; 10 mM) were added, followed by the addition of 400 μL of phosphate buffer solution (20 mM; pH 7.4). For the determination of the non-site-specific assay, the same protocol described above was used, but with the addition of 100 μL of ethylenediaminetetra-acetic acid (EDTA; 1 mM). After incubation for 1 h at 37 °C, 1.5 mL of a 5% TBA solution (thiobarbituric acid, prepared in trichloroacetic acid, 10%) were added. The mixture was boiled (100 °C) for 15 min and the absorbance was read at 532 nm. A reference blank was used as control, using the same protocols but replacing the *T. zygis* solution with 0.5 mL of distilled water. The site-specific and non-site-specific ^•^OH scavenging activity was expressed as the percentage inhibition using Equation (1):

(1) $$Inhibition\;\left(\%\right)=\frac{\mathrm{Blank}\;\mathrm{abs}-\mathrm{Sample}\;\mathrm{abs}\;}{\mathrm{Blank}\;\mathrm{abs}\;}\times 100$$

#### 2.7.3. Nitric Oxide Radical Scavenging Assay

For the determination of the nitric oxide radical (NO^•^) scavenging activity, the method described by Sreejayan and Rao [33] was performed. For the production of the NO^•^, a 5 mM sodium nitroprusside solution in phosphate buffer (0.1 M H_3_PO_4_; pH 7.4) was oxygenated by purging with air for 15 min. To 0.5 mL *T. zygis* extracts (1 mg/mL), 4.5 mL of sodium nitroprusside solution were added and the mixture was incubated for 2 h at 35 °C. NO^•^ was quantified using the Griess colorimetric assay. To 1 mL of the previous mixture, 1 mL of Griess reagent (equal volumes of 1% sulfanilamide in 5% phosphoric acid and 0.1% *N*-alpha-naphthyl-ethylenediamine in water) was added, and the mixture was incubated for 3 min at room temperature. The absorbance was measured at 546 nm. Sodium nitrite was used as the positive control and the NO^•^ scavenging activity was expressed as the inhibition percentage and calculated according to Equation (1). For the blank determination, the *T. zygis* extract solution was replaced by the same volume of distilled water. 

### 2.8. Determination of the Phenolic Profile by High Performance Liquid Chromatography with Diode Array Detector and High Performance Liquid Chromatography with Electrospray Ionization and Tandem Mass Spectrometry Detection

Reversed phase HPLC-DAD analysis was carried out using an Ultimate 3000 HPLC equipped with an Ultimate 3000 pump, a WPS-3000 TSL Analyt auto-sampler and an Ultimate 3000 column compartment coupled to a PDA-100 photodiode array detector (Dionex, Sunnyvale, CA, USA) and HPLC-ESI-MS^n^ analysis was carried out using a Thermo Scientific system equipped with a Finnigan Surveyor Plus auto-sampler, photodiode array detector and pump, and an LXQ Linear ion trap detector was used for LC-MS^n^ analysis as previously described by Taghouti, Martins-Gomes, Schafer, Felix, Santos, Bunzel, Nunes and Silva [32].

Individual phenolic compounds were identified based on ultraviolet-visible (UV-Vis) spectra, retention time, and mass spectra and compared to commercial standards and/or literature data. The calibration curves of available commercial standards were prepared for the quantification of individual phenolic compounds [32]. When no commercial standards were available, phenolic compounds were quantified using the aglycones or standard compounds with structural similarity. Apigenin-(?)-*O*-hexuronide was quantified as apigenin; luteolin-(?)-*O*-hexoside and luteolin-(?)-*O*-hexorunide were quantified as luteolin; salvianolic acid K was quantified as rosmarinic acid.

### 2.9. Quantification of Oleanolic Acid (OA) and Ursolic Acid UA) in Hydroethanolic Extracts

For the quantification of ursolic acid (UA) and oleanolic acid (OA) in the HE extracts, the RP-HPLC (ACE 5 C18 column; 250 mm × 4.6 mm; particle size 5 μm) method, described in [27], was used. The separation was performed using sodium phosphate buffer (30 mM, pH 3) as solvent A, and methanol as solvent B, and during the run, the temperature was held at 40 °C. The identification of OA and UA was performed by UV-VIS spectra (200 to 400 nm) and the retention time of the commercial standards. Quantification was performed using the calibration curves of the UA and OA commercial standards.

### 2.10. In Vitro Cell-Based Assays

#### 2.10.1. Cell Maintenance and Handling

In this study, two human cell lines: Caco-2 (human colon adenocarcinoma cell line; Cell Lines Service, Eppelheim, Germany) and HepG2 (human hepatocellular carcinoma cell line; ATCC^®^ Number: HB-8065TM, a gift from Prof. C. Palmeira CNC-UC, Portugal) and a mouse cell line: RAW 264.7 (mouse macrophages, Abelson murine leukemia virus-induced tumor cell line; Cell Lines Service, Eppelheim, Germany) cells were used to evaluate the anti-proliferative and anti-inflammatory activities of *T. zygis* extracts. Cells were cultured in complete culture media (Dulbecco’s Modified Eagle Media (DMEM), supplemented with 1 mM L-glutamine, 10% (*v*/*v*) fetal bovine serum (FBS), and antibiotics (penicillin at 100 U/mL, and streptomycin at 100 μg/mL) and maintained in incubator (5% CO_2_/95% air; 37 °C, controlled humidity). Near-confluence, Caco-2 and HepG2 cells were sub-cultured by using an enzymatic (trypsin-EDTA) treatment (for 8 and 6 min, respectively for Caco-2 and HepG2 cells), which was stopped using the complete culture medium (1:1, trypsin:culture media), or in the case of RAW 264.7 cells, which were scratched off from the flasks using a cell scratcher (Orange Scientific; Braine-L’Alleud, Belgium). In both cases, cells were gently re-suspended using a Pasteur pipette, counted using an automated cell counter (TC10™, BIORAD, Portugal), and then re-suspended in fresh culture media to achieve a final density of 5 × 10^4^ cells/mL. Cells were then seeded into 96-well microplates (100 µL/well; of 5 × 10^4^ cells/mL), maintained in an incubator, and allowed to adhere and stabilize for 48 h, for other details see [34,35,36].

#### 2.10.2. Cell Viability/Cytotoxicity or Anti-Proliferative Activity Assay

The Alamar Blue assay^®^ [35] was used to assess the anti-proliferative effect of the extracts. Stock solutions (10 mg/mL) of *T. zygis* extracts were prepared in PBS for the AD extract, and 10% DMSO (in PBS) for the HE extract. The DMSO final concentration, in test solutions, was never higher than 1%. After the cell adherence and stabilization period, culture media was removed and replaced with test solutions (100 μL/well) and prepared by dilution of respective stock solutions in FBS-free culture medium (range 50–750 μg/mL for Caco-2 and HepG2, and 10–200 μg/mL for RAW 264.7). After 24 h or 48 h of the cell’s exposure to extracts, test solutions were removed (by gently pipette aspiration), and immediately replaced by Alamar Blue solution (100 μL/well; at 10% (*v*/*v*), in FBS-free culture medium). Absorbance was read, after 5 h incubation, at 570 nm (resorufin; reduced form) and 620 nm (resazurin; oxidized form) using a microplate reader (Multiskan EX; MTX Lab Systems, Inc., Bradenton, FL, USA). In each assay, a control was performed, consisting of non-treated cells (positive control) and Alamar Blue solution alone (negative control). In the positive control, cells were submitted to all procedures (i.e., replacing of media (with only FBS-free culture media), Alamar Blue solution exposure) simultaneously with the cell’s exposure to the test solutions. The results are expressed as cell viability (% of control; i.e., positive control), calculated as described by Andreani, et al. [35].

#### 2.10.3. Anti-Inflammatory Activity

In this work, RAW 264.7 cells were used to assess the anti-inflammatory activity of *T. zygis* extracts, as described by Carbone et al. [34]. Briefly, RAW 264.7 cells previously seeded in 96-well plates (100 µL/well, at 5 × 10^4^ cells/mL), with a stabilization period of 48 h after seeding, were incubated with various concentrations of non-cytotoxic *T. zygis* extract concentrations (see results) in the presence and in the absence of lipopolysaccharide (LPS; at 1 µg/mL). LPS induces the production of nitric oxide (NO) that is released into the incubation media. After incubation (24 h) with extracts from each well, 50 µL of each well supernatant was transferred into a new 96-well plate and, subsequently, 50 µL of Griess reagent [1% (*w*/*v*) sulfanilamide prepared in 5% (*w*/*v*) H_3_PO_4_ (*v*/*v*) and 0.1% (*w*/*v*) *N*-(1-naphthyl) ethylenediamine dihydrochloride in water] were added to each well. After 15 min of incubation time (room temperature, under dark), the absorbance at 550 nm was read (Multiskan EX microplate reader; MTX Lab Systems, Inc., Bradenton, FL, USA). Quantification was performed against a standard curve calculated with sodium nitrite (NaNO_2_; in the range 0 to 100 µM) and the results were expressed as % of control (i.e., nitrite production by the positive control cells (LPS-stimulated cells in the absence of *T. zygis* extracts) set to 100%, that is, 0% of anti-inflammatory effect.

### 2.11. Data and Statistical Analysis

For each extraction method, three individual extractions were performed, and the analyses were performed in triplicate for all the assays. The IC_50_ values for the anti-proliferative activity were calculated as described by Silva et al. [37]. Significant differences for the phenolic composition and antioxidant activity were performed using the t-Student test (α = 0.05). For the comparison of the IC_50_ values for the anti-proliferative activity and anti-inflammatory activity, analyses of variance (ANOVA) followed by Tukey’s multiple test (α = 0.05) were performed (GraphPad Prism version 7, GraphPad Software Inc., San Diego, CA, USA).

## 3. Results and Discussion

### 3.1. Extract Yield and Chemical Composition of T. zygis Extracts

In this study, two extraction methods were used to obtain *T. zygis* subsp. *zygis* (*T. zygis*) extracts: an exhaustive hydroethanolic (HE) extraction and the aqueous decoction (AD). The HE was previously shown to extract 99% of the total extractable compounds [27], thus it was chosen as the method to obtain the full “free” phenolic composition of *T. zygis* subsp. *zygis*. The AD extraction mimics the common procedure of beverage preparation. Therefore, it allows to analyze the phenolic compounds that are available with a common preparation for human consumption, as these plants are also used as herbal teas, seasoning, and condiments. 

Table 1 shows that the yield of *T. zygis* subsp. *zygis* AD extract is higher than the one of the HE extract (~28% higher, *p* < 0.05), denoting differences in the extraction yield between the HE and AD extraction methods. Concerning the AD extract, the yield obtained in this work (29.70 ± 0.99 %, Table 1) is higher than that described for *T. zygis* (subspecies not mentioned) by Afonso et al. (2018), who reported an extraction yield of 12%. Nevertheless, the extraction conditions were significantly different (5 g plant/100 mL of water, for 15 min) from that used in this work. The higher yield values of *T. zygis* extracts (Table 1), compared to other species produced in the same place (such as *Thymus carnosus* [27], *Thymus pulegioides* [32], *T. mastichina* [38] and *T. vulgaris* [39]), might result from a species effect or from the time of year in which they were harvested, as *T. zygis* was harvested in April (blooming stage) and the other ones in October (post-blooming, end fructification stage), the latter hypothesis still needs to be confirmed with more experimental data and other *Thymus* species harvested in the same place at both stages. 

*T. zygis* subsp. *zygis* TPC contents obtained with the HE extraction method were significantly higher than that extracted with the AD extraction procedure (Table 1). The HE extract yielded about twice the TPC contents of the AD extract (HE: 195.81 ± 7.07 and AD: 97.31 ± 7.67 mg CA eq./g extract); but we also observe that, although not exhaustive, AD extraction extracts about 65% of plant’s TPC (Table 1; HE: 44.70 ± 1.61 and AD: 28.90 ± 2.28 mg CA eq./g D.P.).

Comparing the TPC content per gram of dry plant, in plants collected in the same place and extracted with the same HE extraction method, we observed an order for TPC contents (in mg CA eq./g D.P.), *T. carnosus* (84.4 [27]) >> *T. zygis* subsp. *zygis* (44.7, Table 1) ~ *T. pulegioides* (43.0 [32]) >> *Thymus citriodorus* (27.7 [39]) ≥ *T. vulgaris* (25.12 [39]) ~ *T. mastichina* (24.6 [38]). Concerning the AD extraction, the same trend was observed, being the TPC contents (in mg CA eq./g D.P.) of *T. carnosus* (54.5 [27]) >> *T. zygis* subsp. *zygis* (28.9, Table 1) > *T. pulegioides* (26.1 [32]) > *T. vulgaris* (21.6 [39]) >> *T. citriodorus* (15.5 [39]) > *T. mastichina* (12.5 [38]). Methanolic extracts of *T. zygis*, harvested in several locations in Spain, were also reported to have high TPC contents [17] in identical amounts as the here reported (Table 1). These data indicate that *T. zygis* is a suitable source of phenolic compounds.

For the TFC extracted from *T. zygis* subsp. *zygis*, the results obtained are in line with those described for the TPC. The amount of TFC extracted by HE extraction was significantly higher than those obtained by AD extraction (Table 1). For the ODP, as observed for the TFC and TPC, the levels present in the HE extract were significantly higher than that obtained in the AD extract (Table 1). These data highlight the value of the *T. zygis* subspecies *zygis* as a thyme species with high content in potential bioactive molecules.

### 3.2. T.zygis subps. zygis Aqueous Decoction and Hydroethanolic Extracts Phenolic Profiles

**Figure 1:**
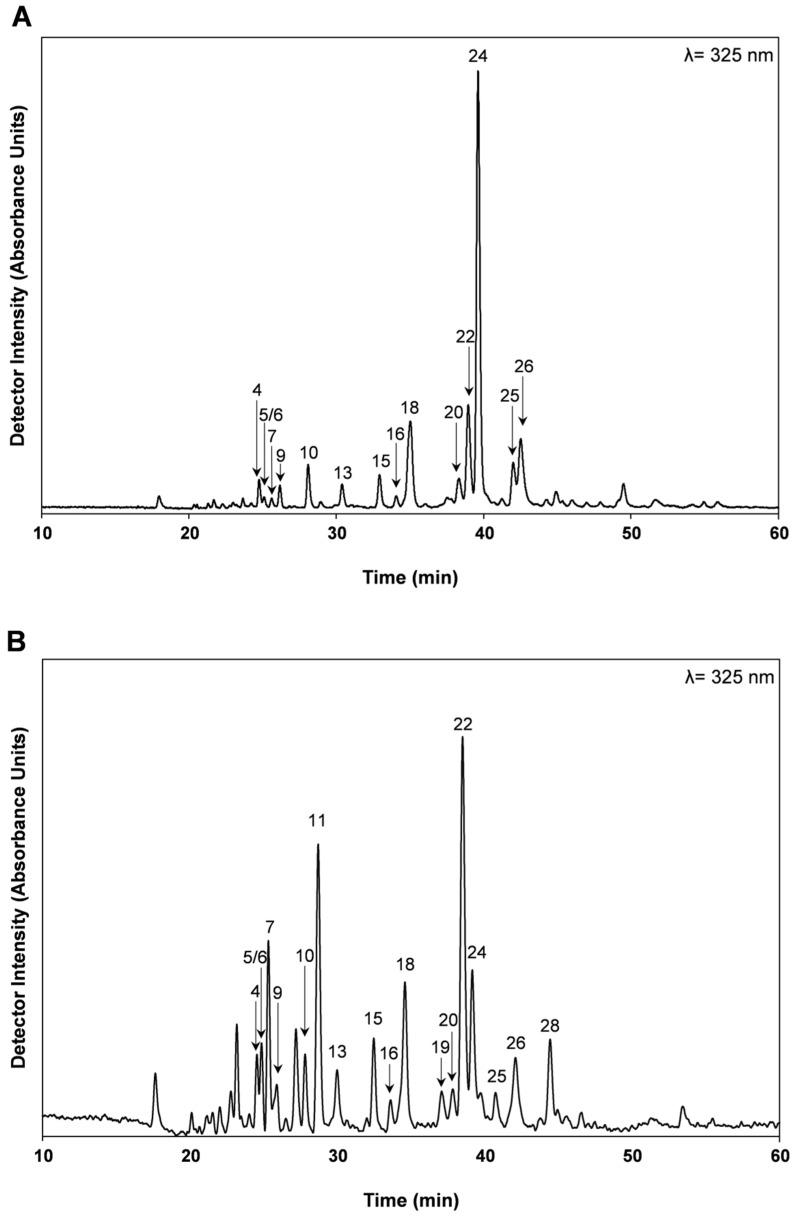
Phenolic profile of hydroethanolic (HE) (**A**) and aqueous decoction (AD) (**B**) extracts of *Thymus zygis* subsp. *zygis*, obtained by High-performance liquid chromatography, coupled to diode array detector (HPLC-DAD). For peak number identification, please refer to Table 2.

In order to have a deeper understanding of the chemical composition of *T. zygis* subsp. *zygis* and the relation with its extracts bioactivities, the phenolic composition of HE and AD extracts was determined by HPLC-DAD and HPLC-MS^n^. The phenolic profile of the HE and AD extracts, as well as their concentrations, is shown in Figure 1 and in Table 2.

The relative amount of phenolic compounds determined by HPLC-DAD is consistent with the results obtained by colorimetry, and with the TPC, TFC, and OPD contents (Table 1). For the HE extracts of *T. zygis* subsp. *zygis*, rosmarinic acid was the most abundant phenolic compound (Table 2). Rosmarinic acid represented 38% of the total phenolic compounds extracted by the HE solution. In the AD extract, the most abundant polyphenol was luteolin-(?)-*O*-hexoside (compound 18; Table 2), accounting for 22% of the total phenolic compounds extracted, followed by rosmarinic acid (21%), and luteolin-(?)-*O*-hexuronide (15%). The amount of rosmarinic acid extracted by AD method represents only 38% of the rosmarinic acid extracted by HE. *Thymus* species are usually characterized by high content of rosmarinic acid [40]. Taking into account that the exhaustive HE extraction method [27] reflects the plant’s total extractable phenolic composition, we observe that *T. zygis* subsp. *zygis* is also characterized by high contents of rosmarinic acid (Table 2). The HE extracts of other *Thymus* species also revealed high contents of rosmarinic acid (as % of total phenolic acids), as is the case of *T. citriodorus* (51% [39]), *T. mastichina* (33% [38]), *T. vulgaris* (70% [39]), *T. pulegioides* (48% [32]). In contrast, other species, such as in *T. carnosus* contain lower amounts of rosmarinic acid (17% [27]). Rosmarinic acid was also quantified in high amounts in aqueous extracts of *T. zygis* (52%, subspecies not mentioned; [19]), [16]) and in *Thymus algeriensis* (45% [41]). Rosmarinic acid was indicated as the major phenolic compound in methanolic extracts of *T. zygis* (subspecies not mentioned; [16]). This *Thymus* differs from the previously mentioned *Thymus* species by the presence of significant amounts of flavonoids (42% and 57% of the total phenolic compounds in the HE and AD extracts, respectively), especially Luteolin-(?)-*O*-hexoside that represents 15% of the total phenolic compounds of the HE extract, was present in lower amounts in *T. vulgaris*, *T. citriodorus*, *T. carnosus*, *T. pulegioides* and *T. mastichina* [27,32,38]. In fact, *T. zygis* subspecies *zygis* is the *Thymus* species studied by our group that contains the second highest levels of flavonoids quantified in the HE extracts (*T. pulegioides* (61%), *T. mastichina* (39%), *T. citrodorus* (24%), *T vulgaris* (16%), and *T. carnosus* (6%)). The AD extraction allowed to recover higher amounts of caffeic acid when compared to the HE extraction. This higher amount of caffeic acid in the AD extraction can be due to the hydrolysis of rosmarinic acid during the AD extraction that is performed with hot water. This hypothesis is supported by the fact that in the AD extracts higher amounts of salvianic acid A were also present (Table 2).

The most abundant phenolic compounds described for *T. zygis* subsp. *zygis* (Table 2) are in agreement with those described by Afonso et al. (2018) for aqueous extracts of *Thymus zygis* (harvested in the same location), although the relative amounts found were different, which might have resulted from extraction procedure (different from the ones in current work), harvesting period, or the use of a different *Thymus zygis* subspecies (not disclosed).

### 3.3. Oleanolic Acid and Ursolic Acid Contents

**Figure 2:**
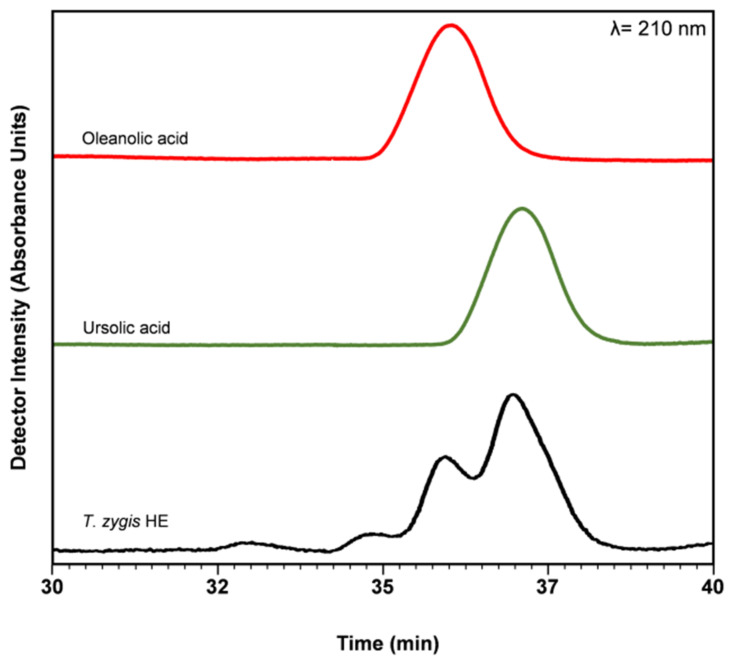
Chromatogram of oleanolic acid (OA) and ursolic acid (UA) standards and of *Thymus zygis* hydroethanolic (HE) extract.

*T. zygis* subsp. *zygis* HE extracts contained OA and UA (Table 2), however, their levels were low (0.26 and 0.55 mg/g dry plants) compared to other thyme species (*T. serpyllum* (3.7 and 13.9 mg/g dry plant, or OA and of UA, respectively [42]), *T. carnosus* (9.9 and 18.7 mg/g dry plant, of OA and of UA respectively [27]) and *T. pulegioides* (0.34 and 0.80 mg/g dry plant, of OA and of UA, respectively [32]). These differences may reflect a different phenological state of the plant, the effect of location, and of the climate on the chemical composition of the plants. To the best of our knowledge, this is the first report in which UA and OA are described in *T. zygis* extracts. However, the presence of the diterpene carnosic acid was described in extracts of *Thymus zygis* subsp. *gracilis* shrubs (cultivated in Spain), obtained with petroleum ether and methanol, in amounts of ~120 µg/g dry plant [18]. The chromatogram of *T. zygis* subsp. *zygis* HE extract, shown in Figure 2, reveals the presence of OA and UA, as compared by the chromatograms of OA and UA standards (two upper traces, as denoted).

### 3.4. In Vitro Antioxidant Activity

The HE extracts of *T. zygis* subsp. *zygis* (at 1 mg/mL) showed higher ABTS^+•^ radical scavenging activity (~0.25 Trolox eq./g dry plant, Table 1) in relation to the AD extracts (~0.23 mmol Trolox eq./g dry plant, Table 1). The values obtained for the HE extracts (Table 1) were lower than those found for *T. pulegioides* HE extracts (0.34 mmol Trolox eq./g D.P.; [32] but in the same range of *T. vulgaris* and *T. citriodorus* (0.22 mmol Trolox eq./g D.P.; [39]), *T. carnosus* (0.21 mmol Trolox eq./g D.P.; [27]) and *T. mastichina* (0.20 mmol Trolox eq./g D.P.; [38]). In contrast, the ABTS^•+^ radical scavenging activity of AD extracts of *T. zygis* subsp. *zygis* (0.21 mmol Trolox eq./g D.P.; Table 1) was higher than that described for the other *Thymus* species (*T. pulegioides* 0.15 mmol Trolox eq./g D.P. [32]; *T. carnosus* 0.14 mmol Trolox eq./g D.P. [27]; *T. mastichina* 0.08 mmol Trolox eq./g D.P. [38] and *T. citriodorus* (0.11 mmol Trolox eq./g dry plant [39]), being similar to that found for AD extracts of *T. vulgaris* (0.20 mmol Trolox eq./g dry plant; [39]). Relevant antioxidant activity was also reported for water extracts of *T. zygis* (ABTS radical scavenging with IC_50_ = 15.43 μg/mL; subspecies not mentioned) collected in the southeastern Morocco [21] and for petroleum ether/methanolic extract of *T. zygis* subsp. *gracilis* (DPPH radical scavenging with IC_50_ = 3.7 μg/mL) harvested in Murcia, Spain [18]. 

*T. zygis* subsp. *zygis* AD extract (at 1 mg/mL) exhibited a higher non-site-specific inhibition activity when compared to the site-specific inhibition activity (Table 1). In the non-site-specific assay we evaluated the efficiency of the compounds present in the extracts to compete with deoxyribose for ^•^OH radicals that are produced by the Fe^2+^-EDTA chelate. On the other hand, for the site-specific assay, when EDTA is not present in the reaction mixture, the Fe^3+^ can bind directly to deoxyribose and produce ^•^OH. Ribose degradation inhibition, in the absence of EDTA, indicates the iron ion chelating possibility and also the trapping of the ^•^OH radical. Therefore, the competition of the compounds present in the extract for scavenging the ^•^OH seems to be the main mechanism, although compounds present in the extract can also effectively bind the Fe^3+^ ions [43,44]. Compared with other works, an IC_50_ value of 3.7 mg/mL was reported for an aqueous extracts of *T. vulgaris* [45], a value much higher than that described here. On the other hand, Chung et al. [46] reported more than 75% inhibition of ribose degradation for a thyme methanolic extract (1 μg/mL; unspecified species). The HO^•^ radical scavenging values obtained for *T. zygis* subsp. *zygis* AD extracts (Table 1) show that this thyme species has a higher inhibition capacity against hydroxyl radical compared to *T. carnosus* (41% [27]), *T. pulegioides* (34% [32]), *T. mastichina* (49% [38]), *T. citriodorus* (38% [39]) and *T. vulgaris* (10% [39]), when the assay was performed in the presence of EDTA. In the absence of EDTA, the *T. zygis* subsp. *zygis* AD extract presented significant inhibition capacity of the ^•^OH (43%, Table 1), too, similar to that observed for AD extracts of *T. carnosus* (41% [27]) but higher than that observed for *T. pulegioides* (31% [32]), *T. mastichina* (28% [38]), *T. citriodorus* (31% [39]) and *T. vulgaris* (21% [39]) AD extracts. *T. zygis* subsp. *zygis* AD extracts produced an inhibition percentage of the ^•^OH lower than that reported of the methanolic extract of *Thymus dacicus* (50% radical scavenging, at 18.85 μg/mL, [47]). 

Concerning the scavenging of the NO radical, *T. zygis* subsp. *zygis* AD extract (29%, Table 1) showed lower scavenging activity than AD extracts of *T. citriodorus* (41%; [39]), *T. vulgaris* (58%; [39]), *T. carnosus* (42%; [27]), *T. pulegioides* (35.76 %; [32]), and *T. mastichina* (39%; [38]).

### 3.5. Anti-Proliferative Effect of T. zygis subsp. zygis Extracts

**Figure 3:**
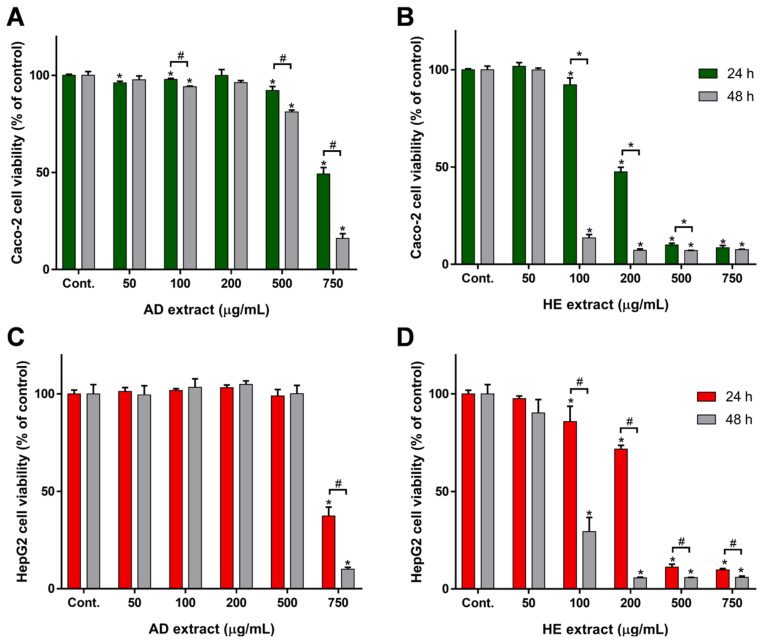
Anti-proliferative effect of *Thymus zygis* subsp. *zygis* aqueous decoction (AD) and hydroethanolic (HE) extracts on Caco-2 (**A** and **B** for AD and HE extracts, respectively) and HepG2 cells (**C** and **D** for AD and HE extracts, respectively). Two exposure times, 24 and 48 h, were considered, as indicated. Results are expressed as (mean ± SD, n = 4). Statistically significant differences (*p* < 0.05) between the control and sample concentrations at respective incubation time are denoted by *, and those between exposure periods at the same concentration are denoted by #.

The anti-proliferative activity of *T. zygis* subsp. *zygis* AD and HE extracts was assessed using the Alamar Blue (AB) assay and the two selected cell lines, HepG2 and Caco-2. Cells were incubated with different concentrations of *T. zygis* subsp. *zygis* extracts (50, 100, 200, 500, and 750 μg/mL) during 24 or 48 h. Results of anti-proliferative assay were compared with positive control cells (non-exposed cells) and are shown in Figure 3. For the AD extract, a reduced anti-proliferative effect was observed in both cell lines (Figure 3A: Caco-2 and 3B: HepG2). As shown, the *T. zygis* subsp. *zygis* AD extract does not reduce HepG2 cells viability for concentrations up to 500 μg/mL (cell viability ~100% of control at 24 and 48 h), however, at 500 μg/mL, it produces a slight reduction of Caco-2 cells viability (cell viability was 92 ± 2% and 81 ± 1%, at 24 and 48 h, respectively). Although, the effect of *T. zygis* subsp. *zygis* AD extract effect is identical on both Caco-2 and HepG2 cells, with close IC_50_ values (Table 3), the differences are statistically different, at both exposure times (*p* < 0.05). 

*T. zygis* subsp. *zygis* HE extract showed a higher cytotoxic/anti-proliferative effect than the AD extract, in both cell lines (Figure 3B: Caco-2 and 3D: HepG2). *T. zygis* subsp. *zygis* HE extract is non-toxic at 50 μg/mL (both cell lines, both exposure times). Using the HE extract at 100 μg/mL, only the 24 h exposure may be considered non-toxic (cell viability was 92.3 ± 3.4% and 85.8 ± 7.9%, for Caco-2 and HepG2, respectively; *p* < 0.05), while the 48 h exposure is toxic (cell viability was 13.6 ± 1.7% and 29.4 ± 9.3%, for Caco-2 and HepG2, respectively; *p* < 0.05). The HE extract at concentrations higher than 200 μg/mL reduces cell viability to values below 20% of control (both cell lines, both exposure times). As the human consumption of this plant (herbal tea, seasoning or condiments) is mimicked by the AD extract effect, the results indicate that *T. zygis* subsp. *zygis* is non-toxic. However, the high anti-proliferative activity/cytotoxicity of HE extracts on Caco-2 cells (IC_50_ 85.01 ± 15.10 µg/mL, at 48 h exposure, Table 3) and on HepG2 cells (IC_50_ 82.19 ± 2.46 µg/mL, at 48 h exposure, Table 3) makes this species a good source for bioactive molecules with anti-proliferative activity. This effect correlates with its higher concentration in phenolic compounds in HE extract (Table 1 and Table 2), and most probably it is due to the presence of UA and OA. The anti-proliferative activity of these triterpenoids against several tumor cell lines is documented in several works [48,49] OA exerted strong cytotoxic effect against HT29 cells (colon adenocarcinoma) with EC_50_ = 5.6 μM [50], and UA and OA exerted significant anti-tumor activity against HCT15 cells (human colon carcinoma cell line) by inhibiting cell proliferation through cell-cycle arrest [49]. Among the *Thymus* species studied in our laboratory, *T. carnosus* contains higher contents of UA and OA in its HE extract [27], and produced lower IC_50_ values in both cell lines (Caco-2 ~32 µg/mL, and HepG2 ~120 µg/mL, at 24 exposure). However, we may not exclude the effect of the other compounds that may synergistically affect this anti-proliferative activity (Figure 3, Table 3). Additionally, rosmarinic acid, the major phenolic acid in this extracts (Table 1) has been widely described to produce anti-proliferative activity in several cell models, by mechanisms that involve apoptosis regulation and cell-cycle arrest in sub-G1 and G2/M phases [51,52]. Salvianolic acids have been widely described to control cellular pathways involved in cell proliferation and in cellular migration, which are intrinsically related to cancer progression [53,54], although most of the reported activities are to salvianolic acids A and B, salvianolic acids K and I (Table 2) might share structurally-related activities. The overall response of cells to *T. zygis* subsp. *zygis* extracts results from the combined activity of all components.

### 3.6. Anti-Inflammatory Effect of T. zygis subsp. zygis Extracts

**Figure 4:**
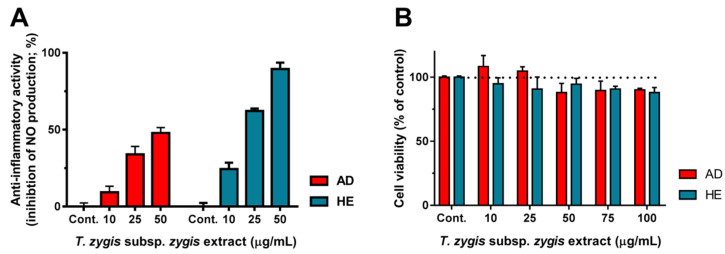
Anti-inflammatory activity of *Thymus zygis* subsp. *zygis* extracts. (**A**) Inhibition of nitric oxide (NO) release by LPS-stimulated RAW 264.7 cells induced by aqueous decoction (AD; left bars, red) and by hydroethanolic (HE; right bars, blue) extracts, expressed as percentage of control (see methods for details). (**B**) Effect of AD (red bars) and HE (blue bars) extracts on RAW 264.7 cells viability (see methods for details). Results are expressed as mean ± SD (n = 4 independent assays).

The anti-inflammatory activity of *T. zygis* subsp. *zygis* was evaluated on RAW 264.7 cells, as a consequence of extracts capacity to decrease the lipopolysaccharides (LPS)-induced nitric oxide (NO) release when exposed to *T. zygis* subsp. *zygis* extracts, and is shown in Figure 4. First, a cell viability assay was performed on RAW 264.7 cells (Figure 4B) to select non-cytotoxic concentrations of *T. zygis* subsp. *zygis* extracts. The cells were exposed to AD and HE extracts, at concentrations up to 100 µg/mL, for 24 h. Figure 4B shows that both extracts are not cytotoxic. Due to the slight decrease in cell viability at 100 µg/mL of extracts (91.2 ± 1.2% and 88.1 ± 3.8%, for AD and HE extract, respectively, not statistically different from control, *p* > 0.05)) the concentrations selected for the anti-inflammatory assay were up to 50 µg/mL (Figure 4A). Both extracts showed a dose-dependent inhibition of NO release by LPS-stimulated RAW 264.7 cells, indicating anti-inflammatory activity. The HE extract resulted in an about two-fold higher effect compared to the AD extract, which might be the result of the higher content in phenolic compounds (Table 1 and Table 2). Anti-inflammatory activity, using the same cell model as in this work, was reported for *T. zygis* subsp. *sylvestris* essential oils [11], too. Using the inhibition of protein denaturation method to estimate anti-inflammatory activity, Hmidani et al. [21] reported good anti-inflammatory activity of *T. zygis* subsp. *gracilis* aqueous extracts (*T. zygis* IC_50_ = 133.25 μg/mL, and indomethacin IC_50_ = 86.07 μg/mL). Using in vivo models, an aqueous extract of *T. zygis* subsp. *gracilis* was reported to cause potent anti-inflammatory activity in the mice model of croton oil-induced ear edema and significant anti-inflammatory activity in the carrageenan-induced paw edema rat model, in comparison with indomethacin [20]. The croton oil-induced ear edema mice model was also used to evaluate the anti-inflammatory effect of *Thymus broussonetii* (in extracts and fractions) revealing that the main anti-inflammatory principles were UA and OA [55]. OA and UA, with a skeleton of oleanane and ursane, are considered the main responsible for the anti-inflammatory activity exhibited by a variety of medicinal plants [56]. This is attributed to the inhibition of enzymes involved in, e.g., eicosanoids production (COX, cyclooxygenase; and phospholipase A2), that results in reducing processes inflammatory [48,57,58,59]. However, the AD extract that did not contain UA and OA also showed significant anti-inflammatory activity. In addition, although, the presence of UA and OA in the HE extract could justify the higher anti-inflammatory activity of the HE extract, compared to the AD extract (Figure 4), the amount of UA and OA at 50 μg/mL of the HE extract was about 0.1 μM (~50 ng/mL), which is very low. However, Figure 4A,B show that, at each tested concentration, the HE extract is about two-fold more potent than the AD extract, which corresponds to the ratio of the total phenolic compounds between the HE and AD extracts (Table 2). Therefore, it can be suggested that other compounds that are present in higher levels in the HE extract may also contribute to the observed potent anti-inflammatory activity of *T. zygis* subsp. *zygis* HE extract [60]. Thus, we may confirm and conclude that phenolic compounds of *T. zygis* subsp. *zygis* have high anti-inflammatory potential.

Concerning the phenolic composition of these extracts and related contribution to the verified anti-inflammatory effect, rosmarinic acid has been demonstrated to produce an anti-inflammatory effect both in in vitro and in vivo experimental models, and by modulating several mechanisms [40]. In RAW 264.7 cells, rosmarinic acid was shown to inhibit inducible nitric oxide synthase (iNOS) activity [61], resulting in lower NO released levels. The reduction of NO release induced by rosmarinic acid by LPS-stimulated RAW 264.7 cells was also reported by Martins-Gomes et al. [27]. Recently, other *Thymus* species, *Thymus algeriensis*, was reported to have a high content in salvianolic acid K, together with rosmarinic acid and luteolin glucuronide, which was correlated with the reported anti-inflammatory activity [62]. The role of the individual phenolic compounds of extracts, such as the salvianolic acid K and I, in modulating specific cellular pathways involved in inflammation and proliferation are worth of further study aiming at the discovery of novel pharmacological relevant molecules.

## 4. Conclusions

To the best of our knowledge, this is the first work describing the detailed phytochemical composition of *T. zygis* subsp. *zygis*, a *Thymus* species endemic of Portugal. When compared to other *Thymus* species, it contains a higher content of luteolin-(?)-*O*-hexoside, a polyphenol present in other *Thymus* species in lower amounts. Furthermore, its AD extract presented high amounts of luteolin derivatives including the luteolin-(?)-*O*-hexoside and luteolin-(?)-*O*-hexuronide. The amount of total phenolic compounds of the *Thymus* species analyzed in this study is comparable to the total phenolic contents of commercial *Thymus* species, namely *T. vulgaris* and *T. citriodorus*. Additionally, *T. zygis* subsp. *zygis* presented a high antioxidant activity against the ABTS radical and OH radical when compared to other *Thymus* species. The AD extract of *T. zygis* subsp. *zygis* showed low anti-proliferative/cytotoxic activity, but HE extracts exhibited high anti-proliferative activity. Additionally, both extracts showed high anti-inflammatory activity, at low concentrations, because they were able to reduce the NO release by LPS-stimulated RAW 264.7 cells.

*T. zygis* subsp. *zygis* has thus a great potential to be used as a functional food, for example as decoction or herbal tea or as condiment. Furthermore, due to the biological activities presented by the phenolic compounds, especially in the HE extract, it can also be a source of bioactive ingredients with antioxidant, anti-proliferative, and anti-inflammatory properties.

## Figures and Tables

**Table 1:** *Thymus zygis* subsp. *zygis* extracts: extraction yields, chemical composition, and antioxidant activity.

		Hydroethanolic Extract	Aqueous Decoction
**Extraction yield** (%, *w*/*w*)		22.83 ± 0.96	29.70 ± 0.99 *
**Total phenols** (mg Caffeic acid eq./g)	**Ext.**	195.81 ± 7.07	97.31 ± 7.67 *
	**D.P.**	44.70 ± 1.61	28.90 ± 2.28 *
**Total flavonoids** (mg Catechin eq./g)	**Ext.**	269.49 ± 10.39	124.80 ± 11.62 *
	**D.P.**	61.52 ± 2.37	37.07 ± 3.45 *
***Ortho-*diphenols** (mg Caffeic acid eq./g)	**Ext.**	139.79 ± 1.28	78.55 ± 0.80 *
	**D.P.**	31.91 ± 0.29	23.33 ± 0.24 *
**ABTS^•+^** (mmol Trolox eq./g)	**Ext.**	1.08 ± 0.15	0.76 ± 0.14
	**D.P.**	0.25 ± 0.03	0.23 ± 0.04
**^•^OH radicals + EDTA** (% inhibition)			66.28 ±1.20
**^•^OH radicals − EDTA** (% inhibition)			43.15 ±2.88
**NO^•^ radicals** (% inhibition, after 120 min)			29.32 ± 1.67

Abbreviations: D.P., dry plant; Ext., extract. In antioxidant activity, the percentage of inhibition was obtained for 1 mg/mL of extract. Significant statistical differences between extraction methods (*) when (*p* < 0.05).

**Table 2:** Phytochemical composition of *Thymus zygis* subsp. *zygis* hydroethanolic (HE) and aqueous decoction (AD) extracts determined by high performance liquid chromatography coupled to diode array detector and electrospray ionization mass spectrometry.

	Compound	R.T. (min)	ESI-MS^2^	Quantification				
				HE		AD		E. M. E.
				mg/g D.P.	mg/g Extract	mg/g D.P.	mg/g Extract	
**1**	Salvianic acid A	18.48 ± 0.22	[197]	n.q.	n.q.	0.17 ± 0.01	0.58 ± 0.4	
**2**	Eriodictyol-di-*O*-hexoside	21.81 ± 0.19	[611]:449;287	n.q.	n.q.	n.d.	n.d.	
**3**	Chlorogenic acid	23.72 ± 0.21	[353]:191;179;173;135	n.q.	n.q.	n.d.	n.d.	
**4**	Unknown	24.72 ± 0.22	[563]:545;517;455	n.q.	n.q.	n.q.	n.q.	
**5**	Hydroxyjasmonic acid–hexoside	24.89 ± 0.10	[387]:369;225;207;163	0.03 ± 0.01	0.14 ± 0.03	0.31 ± 0.01	1.07 ± 0.02	*
**6**	Apigenin-(6,8)-*C*-diglucoside	25.02 ± 0.18	[593]:575;503;473;383 353	0.17 ± 0.04	0.76 ± 0.15	0.56 ± 0.03	1.85 ± 0.1	*
**7**	Caffeic acid	25.52 ± 0.22	[179]:135	0.08 ± 0.02	0.36 ± 0.09	0.56 ± 0.01	1.89 ± 0.02	*
**8**	Unknown	26.05 ± 0.19	[495]:486;451;375;368	n.q.	n.q.	n.q.	n.q.	
**9**	Eriodictyol-(?)-*O*-hexoside	26.16 ± 0.23	[449]:287	2.00 ± 0.26	8.77 ± 1.13	1.43 ± 0.05	4.80 ± 0.17	*
**10**	Unknown	27.95 ± 0.95	[367]:193;173;155;137;111	n.q.	n.q.	n.q.	n.q.	
**11**	Prolithospermic acid	28.73 ± 0.25	[357]:313;269;245;203	n.d.	n.d.	n.q.	n.q.	
**12**	Naringenin-*O*-hexoside	29.4 ± 0.26	[433]:313;271;267;137	n.d.	n.d.	n.q.	n.q.	
**13**	Quercetin-(?)-*O*-hexoside	30.48 ± 0.04	[463]:301	0.92 ± 0.17	4.05 ± 0.76	0.39 ± 0.06	1.31 ± 0.22	
**14**	Naringenin-*O*-hexoside	30.88 ± 0.34	[433]:313;271	n.q.	n.q.	n.q.	n.q.	
**15**	Luteolin-*O*-hexoside	32.74 ± 0.41	[447]:285	0.83 ± 0.12	3.64 ± 0.51	0.70 ± 0.05	2.38 ± 0.16	*
**16**	Luteolin-(?)-*O*-rutinoside	33.87 ± 0.30	[593]:285	n.q.	n.q.	n.q.	n.q.	
**17**	Quercetin-(?)-*O*-hexuronide	34.42 ± 0.28	[477]:301	n.q.	n.q.	n.q.	n.q.	
**18**	Luteolin-(?)-*O*-hexoside	34.84 ± 0.30	[447]:285	4.44 ± 0.57	19.46 ± 2.49	4.23 ± 0.30	14.23 ± 1.00	
**19**	Salvianolic acid B/E isomer 2	37.30 ± 0.28	[717]:555;519;475;357;295	n.q.	n.q.	n.q.	n.q.	
**20**	Quercetin-(?)-acetyl-hexoside	38.06 ± 0.38	[549]:531;505;486;416;345;301	1.03 ± 0.22	4.52 ± 0.87	0.98 ± 0.15	3.30 ± 0.5	
**21**	Salvianolic acid A isomer	38.46 ± 0.34	[493]:383;313;295	n.q.	n.q.	n.d.	n.d.	
**22**	Luteolin-(?)-*O*-hexorunide	38.77 ± 0.34	[461]:285;175	2.92 ± 0.32	12.78 ± 1.40	3.01 ± 0.32	10.14 ± 1.06	
**23**	Chrysoeriol-(?)-*O*-hexoside	39.90 ± 0.33	[461]:299;160	n.q.	n.q.	n.q.	n.q.	
**24**	Rosmarinic acid	39.44 ± 0.38	[359]:223;179;161	11.11 ± 1.39	48.65 ± 5.34	4.18 ± 0.78	14.07 ± 2.62	*
**25**	Salvianolic acid I	41.37 ± 0.88	[537]:493;448;359;339;313;295	3.31 ± 0.48	14.52 ± 2.10	0.95 ± 0.20	3.21 ± 0.65	*
**26**	Salvianolic acid K	42.33 ± 0.30	[555]:537;493;359	2.36 ± 0.37	10.33 ± 1.62	2.11 ± 0.30	7.10 ± 1.03	
**27**	Quercetin-(?)-*O*-hexoside-hexuronide	43.36 ± 0.38	[639]:301	n.q.	n.q.	n.q.	n.q.	
**28**	Apigenin-(?)-*O*-hexuronide	44.73 ± 0.44	[445]:269;175	n.q.	n.q.	n.q.	n.q.	
**29**	Chrysoeriol-(?)-*O*-hexuronide	45.86 ± 0.30	[475]:299	n.q.	n.q.	n.q.	n.q.	
**30**	Unknown	49.50 ± 0.44	[551]:519;359;339;313;221;179	n.q.	n.q.	n.d.	n.d.	
**31**	Oleanolic acid ^a^	35.85 ± 0.05	-	0.22 ± 0.03	0.99 ± 0.15	n.d.	n.d.	
**32**	Ursolic acid ^a^	36.91 ± 0.05	-	0.48 ± 0.08	2.17 ± 0.35	n.d.	n.d.	
			**Total phenolic compounds**	29.22 ± 3.47	127.98 ± 15.20	19.58 ± 2.25	65.93 ± 7.56	*
			**Total phenolic acid**	16.90 ± 2.03	74.00 ± 8.92	8.30 ± 1.29	27.93 ± 4.35	*
			**Total flavonoids**	12.32 ± 1.44	53.98 ± 6.31	11.28 ± 0.96	37.99 ± 3.21	

Abbreviations: AD: aqueous decoction; HE: hydroethanolic extractions; RT: retention time; ESI-MS^2^-Fragment ions obtained after fragmentation of the pseudo-molecular ion [M]^−^; n.q.: not quantified (but detected); n.d.: not detected; E.M.E.: extraction method effect. ^a^—identified and quantified by a different method. (*) denotes significant statistical differences (*t*-Student test), between extraction methods, for mg/g dry plant (D.P.), if (*p* < 0.05). Results, from n = 3 different extractions, per extract, are presented as mean ± standard deviation.

**Table 3:** Effect *Thymus zygis* subsp. *zygis* extracts on Caco-2 and HepG2 cells, expressed as IC_50_ values. Cells were exposed to aqueous decoction (AD) and hydroethanolic (HE).

		IC_50_ (µg/mL)		Exposure Time Effect		Extraction Method Effect
		AD	HE	AD	HE	
**Caco-2**	24 h	746.10 ± 6.35	202.20 ± 5.59	*	*	*
	48 h	604.70 ± 6.70	85.01 ± 15.10			*
**HepG2**	24 h	719.20 ± 8.65	264.90 ± 10.03	*	*	*
	48 h	638.02 ± 5.24	82.19 ± 2.46			*

Results are expressed as (mean ± SD, n = 4); * means statistically significant at *p* < 0.05.
